# Reference Device-Assisted Adaptive Location Fingerprinting

**DOI:** 10.3390/s16060802

**Published:** 2016-06-01

**Authors:** Dongjin Wu, Linyuan Xia

**Affiliations:** School of Geography and Planning, Sun Yat-Sen University, 135 # Xingangxi Road, Guangzhou 510275, China; wudj@mail2.sysu.edu.cn

**Keywords:** adaptive location fingerprinting, interpolation, Universal Kriging, device heterogeneity, differential RSSI

## Abstract

Location fingerprinting suffers in dynamic environments and needs recalibration from time to time to maintain system performance. This paper proposes an adaptive approach for location fingerprinting. Based on real-time received signal strength indicator (RSSI) samples measured by a group of reference devices, the approach applies a modified Universal Kriging (UK) interpolant to estimate adaptive temporal and environmental radio maps. The modified UK can take the spatial distribution characteristics of RSSI into account. In addition, the issue of device heterogeneity caused by multiple reference devices is further addressed. To compensate the measuring differences of heterogeneous reference devices, differential RSSI metric is employed. Extensive experiments were conducted in an indoor field and the results demonstrate that the proposed approach not only adapts to dynamic environments and the situation of changing APs’ positions, but it is also robust toward measuring differences of heterogeneous reference devices.

## 1. Introduction

The explosive growing of location-based services (LBS) has brought high demands for location information, and fostered research interest in positioning technology. Besides Global Navigation Satellite Systems (GNSS), indoor positioning technology has gained much attention. Existing wireless infrastructures such as WiFi access points (AP), that are designed for ubiquitous network access and communication, show great potential for providing wireless positioning and augmenting GNSS indoors.

Received signal strength indicator (RSSI)-based location fingerprinting approaches are often adopted in indoor wireless positioning systems. Although the construction of radio maps involves great labor costs, location fingerprinting can provide more accurate estimations compared to other techniques, such as Cell-ID and trilateration, especially in dynamic environments. Location fingerprinting involves two steps: radio map construction and location estimation. The radio map is a combination of signal strength profiles of the APs that cover the target area. It is always approximated by discrete location fingerprints collected at predefined calibration points. Based on the constructed radio map, online RSSI measurements are matched to find the most similar location fingerprints which are further used for location estimation [1]. For practical use, constructing a radio map is a burdensome task. Even worse, the radio map needs continuous updating to accommodate the changing environments. Environmental dynamics, such as people moving, and doors opening and closing can cause rapid RSSI fluctuations. Moreover, the movement of APs or furniture will change the RSSI profiles. All the mentioned factors can lead to an outdated radio map which deteriorates the location fingerprinting performance and therefore repeated site surveys or calibrations are needed from time to time. As a result, approaches that adapt to dynamic and complex environments are more applicable [2,3,4,5,6,7,8,9]. However, heterogeneity [10] of reference devices is an additional issue as for adaptive approaches which always rely on real-time or online RSSI samples measured by a group of reference devices or users’ devices. Unfortunately, the issue of reference device heterogeneity has not been addressed in former adaptive approaches. In this paper, we propose an approach that adapts to dynamic and complex environments and is robust with respect to reference device heterogeneity. Based on real-time RSSI samples measured by a group of reference devices, a modified Universal Kriging (UK) algorithm is applied to estimate radio maps. In addition, a differential RSSI metric is applied to alleviate the impacts of reference device heterogeneity. The contributions of our work can be summarized as follows:

- A modified UK method that builds semivariograms relying on a novel way is proposed for the estimation of RSSI. The modified UK-based adaptive location fingerprinting approach can provide better location performance, compared with the conventional UK-based approach.
- Compared with other adaptive techniques, our proposed approach can not only adapt to dynamic environments, but also accommodate to changing AP positions.
- The issue of reference device heterogeneity in adaptive approaches is addressed for the first time. A differential RSSI metric is verified to be appropriate to alleviate the impacts caused by heterogeneous reference devices.

The rest of this paper is organized as follows: Section 2 presents related work on adaptive approaches for radio map construction and methods for handling device heterogeneity, whereas Section 3 introduces the proposed adaptive location fingerprinting method. Experiments and results are shown in Section 4. Section 5 draws the conclusions.

## 2. Related Work

### 2.1. Adaptive Approaches for Radio Map Construction

Dynamic environments have been widely considered in research on wireless positioning. To obtain robust estimates of RSSI, mean or median sample values are applied. However, these estimates that rely on samples from a short time period cannot represent exactly the long term characteristics of RSSI measurements, not to mention the fact that RSS samples may have complex distributions during different time periods. For example, RSS values at a sampling point sometimes approximate a Gaussian distribution [11]. However, the distributions are always skewed [12], and sometimes with a long tail [13]. Besides, two peak distribution [14] and multimodal distribution [12] are also found. To overcome the limitations of samples over specific time periods, and to reduce the heavy data collection burden, crowd-sourcing-based methods [15,16] or reference device-based methods [2,3,4,5,6,7,8,9] provide alternatives. Our work focuses on the reference device-based manner. On the one hand, RSSI measurements can be mapped to geographical distances [2], and trilateration is then applied to estimate locations. On the other hand, RSSI measurements can be used to estimate the signal strength profiles of APs [3,4,5,6,7,8,9], and location fingerprinting is then used for location estimation. In this paper, the discussions follow the latter approach. Although we can place one reference device at each predefined point just as the LANDMARC system does [6], the method relies on too many reference devices. Applying interpolation methods can effectively reduce the number of reference devices. For example, in the LEASE system [7], the Akima Spline interpolation method is used to estimate radio maps. Besides, other interpolation methods, such as the inverse distance weighting (IDW) [17,18], radial basis function (RBF) [19], linear [18,20], and Kriging ones [21,22,23] can be applied to generate radio maps too. Before applying these interpolation methods, we should learn the characteristics of each one and make them suit the estimation of RSSI. For example [24], the IDW method estimates the values at unsampled points using a linear combination of values at sampled points weighted by an inverse value of the distance from the unsampled point to the sample points. The assumption is that the closer the distance is, the more similar are the values between the unsampled point and the sample point. The nearest neighbours method predicts the value at an unsampled point based on the value of the nearest sample. Among these methods, the Kriging methods first employ the idea of geostatistics that besides the relative positions between the unsampled point and the sample points, additionally the spatial correlation of the variables is considered.

Unlike the above conventional interpolation methods, radio signal propagation model-based interpolation methods take the characteristics of radio signals into account and therefore to some extent can provide more accurate RSSI estimates. For example, Atia *et al.* [8] proposed a solution that is based on online RSSI samples from the existing WLAN APs which have been modified as a combination of reference devices and conventional APs. In the solution, the Gaussian Process Regression (GPR) method was used to augment the Log distance path loss model to obtain AP signal strength profiles. Similarly, modified APs are also applied by Du *et al.* [9]. With additional self-made sensor anchors, they use the Geography Weighted Regression (GWR) method to obtain a more accurate signal propagation model to construct radio maps. The so called RSSI Geography Weighted Regression (RGWR) is a variable-coefficient regression method that maintains as a significant feature the fact that signals attenuate with different characteristics in different regions. In order to apply the RGWR method, the coordinates of APs and anchors, as well as the distances between each pair of anchor and AP are necessary, besides signal strength information. For the above two approaches, although the modified APs can be replaced by reference devices and conventional APs, APs’ positions are required, which brings additional information to manage and are unpractical for random APs that are much more numerous than deployed APs. Based on the Log distance path loss propagation model, Lee *et al.* [25] also proposed to refine the model for each partitioned cell depending on a high order Voronoi diagram which is generated according to the reference points. In their method, APs’ positions are estimated simultaneously when estimating the parameters of the propagation model. More than a radio signal propagation model, the RSSI regression relationship between the sampling points of the radio map and neighboring reference devices can be obtained and utilized too. New radio maps can be estimated based on the regression relationship [3,4,5]. For example, Yin *et al.* [3] used a decision tree to model the relationship. According to the deployment of all reference devices, the reference-point value space is partitioned into several regions. An assumption is that the RSSI relationship between reference devices and the mobile client is linear when they are in the same region. With the trained model, radio maps can be updated effectively even with one reference device. Wang *et al.* [4] proposed to use neural networks to build the relationship. In their work, a three-layer structure is applied, and data from all the reference devices are used as the inputs, whereas data at each sampling point is used as the output. Although these methods perform well in time-varying environments, the assumption of invariable RSSI regression relationships between a sampling point and the neighboring calibration points is not true once some APs’ positions are changed.

### 2.2. Methods for Handling Device Heterogeneity

None of the former adaptive approaches solves the issue of device heterogeneity which prevents those approaches from being practically applied. Since receivers with different hardware and software measure totally differently from each other, it is necessary to compensate the differences because the devices that are used for location estimation are various, such as notebooks, smart phones, *etc.* The solutions for tackling device heterogeneity can be divided into two categories: creating a robust metric and mapping the original values to a standard value space [26]. Depending on the observations, RSSI samples measured by two different devices at the same place have a consistent relationship [27,28,29]. Thus RSSIs measured by different devices can be mapped to reasonable values based on the relationship. First a linear relationship [27] is found between the RSSI measurements of two devices. Although manual calibration always produces a large improvement in terms of location accuracy [28], it is tedious since every device needs calibration. As a result, automatic calibration has drawn more attention. For example, based on a linear relationship, Haeberlen *et al.* [28] investigated three manners—manual, quasi-automatic and automatic calibration—to approximate the model. Tsui *et al.* [29] proposed an unsupervised learning method to automatically obtain a transformation function for mapping WiFi signal patterns from an unknown device to the training device. Experimental results show that position error caused by device heterogeneity is reduced about 4%. However, these methods cannot work without a learning procedure, and they can only work in pairwise mode. Both of these characteristics limit their usability. On the contrast, robust metrics can save the learning step and break the pairwise mode. Differential RSSI [15,30,31,32,33,34,35] is believed to be effective since it eliminates the antenna gains of receivers from a theoretical view [30], but it uses one dimension less than original RSSI vector [31] and may create a greater range of locations that satisfy a given sample set of RSSI signals [32]. The form of the differential RSSI vector is presented in [33], and there are $\left(\begin{array}{c} N\\ 2 \end{array}\right)$ elements in the vector (*N* denotes the number of the elements in the original RSSI vector) in which only (*N* − 1) of the elements are independent. Besides differential RSSI, other metrics were also applied in previous studies. For example, a method named hyperbolic location fingerprinting (HLP) based on RSSI ratio is proposed in [36]. Although the proposed RSSI ratio is observed to be more stable than absolute values, there is no theoretical analysis on the reason why it mitigates the measuring differences of heterogeneous devices. Cheng *et al.* [37] proposed a combined metric, the ratio of signal strength differences which eliminates measuring differences caused by device heterogeneity and dynamic environments. However, this approach needs RSSI readings from three closely located antennas which are usually unavailable for common users’ devices.

## 3. Reference Device-Assisted Adaptive Location Fingerprinting

**Figure 1:**
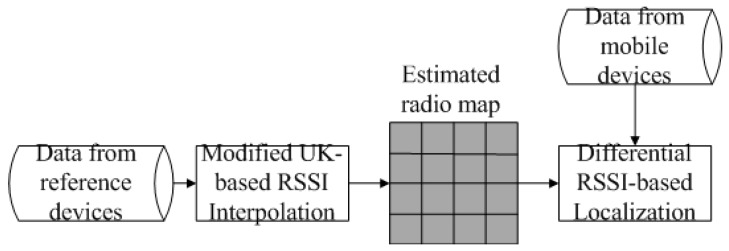
Flow chart of reference device-assisted adaptive location fingerprinting.

Reference device-assisted adaptive location fingerprinting approach consists of two steps: radio map construction and location estimation. However, the two steps are both performed online, which is different from conventional location fingerprinting. The following subsections present the approach in detail. First, a modified UK interpolation is proposed for the interpolation of RSSI. Second, a differential RSSI metric is adopted and deduced for multiple reference device-based location estimation. The flow chart of the proposed approach is depicted in Figure 1.

### 3.1. Radio Map Construction

Radio signals have two inherent characteristics: large-scale attenuation and small-scale fading [38]. Large-scale attenuation indicates the impacts of the travel paths of signals. Small-scale fading refers to the impacts of local environmental dynamics. The fluctuations caused by the small-scale fading are always time-varying, and they are highly correlated with human activities [5]. For example, during work hours, RSSI values fluctuate more rapidly, and if offline calibration is conducted during this time period, it will be unsuitable for online use during non-work hours. Thus we must construct suitable radio maps for location estimation. Radio maps estimated by using real-time RSSI measurements can be the candidates for this.

#### 3.1.1. RSSI Estimation by Using the UK [39] Method

To construct a radio map which is a combination of location fingerprints, RSSI estimates with respect to each AP at predefined points should be obtained first. In this paper, we choose UK as the primary estimation method, and propose a novel way of obtaining the semivariogram models accommodating the characteristics of RSSI spatial distribution.

Kriging interpolants are but variants of the linear estimator [40]:

(1) $$\hat{Z}\left(x_{0}\right)-m\left(x_{0}\right)=\sum_{i=1}^{n}\omega_{i}\left[Z\left(x_{i}\right)-m\left(x_{i}\right)\right]$$

where $m\left(x_{0}\right)$ and $m\left(x_{i}\right)$ are expected values of $Z\left(x_{0}\right)$ and $Z\left(x_{i}\right)$. *x*_0_ is the unsampled point, and {*x_i_*} are the neighboring sampled points. $\omega_{i}$ is the weight of sampled point *x_i_*. $Z\left(x_{0}\right)$ is treated as a random field with a trend component, $m\left(x_{0}\right)$ and a residual component, $R\left(x_{0}\right)=Z\left(x_{0}\right)-m\left(x_{0}\right)$. For RSSI, $m\left(x_{0}\right)$ is not just a constant but a linear or higher-order trend in the (x, y) coordinates of the data point. Assume that:

(2) $$m\left(x\right)=\sum_{k=1}^{p}\beta_{k}s_{k}\left(x\right)$$

where, $s_{k}\left(\cdot \right)$ is a function of location, $\beta_{k}$ is an unknown parameter. This kind of Kriging with a trend is known as UK.

Substituting Equation (2) into Equation (1), we get:

(3) $$\hat{Z}\left(x_{0}\right)=\sum_{i=1}^{n}\omega_{i}Z\left(x_{i}\right)+\sum_{k=1}^{p}\beta_{k}s_{k}\left(x_{0}\right)-\sum_{i=1}^{n}\omega_{i}\sum_{k=1}^{p}\beta_{k}s_{k}\left(x_{i}\right)$$

We require:

(4) $$\sum_{k=1}^{p}\beta_{k}s_{k}\left(x_{0}\right)-\sum_{i=1}^{n}\omega_{i}\sum_{k=1}^{p}\beta_{k}s_{k}\left(x_{i}\right)=0$$

leading to an UK estimator:

(5) $${\hat{Z}}_{UK}\left(x_{0}\right)=\sum_{i=1}^{n}\omega_{i}^{UK}Z\left(x_{i}\right)$$

with:

(6) $$\sum_{k=1}^{p}\beta_{k}s_{k}\left(x_{0}\right)=\sum_{i=1}^{n}\omega_{i}\sum_{k=1}^{p}\beta_{k}s_{k}\left(x_{i}\right)$$

For simplification, we require:

(7) $$\sum_{i=1}^{n}\omega_{i}s_{k}\left(x_{i}\right)=s_{k}\left(x_{0}\right),k=1,...,p$$

Note that we can still hold the constraint Equation (7). Thus we have a minimization problem with *p* constraints that can be solved using Lagrange multipliers, *λ_k_*:

(8) $$L=\sigma_{E}^{2}\left(x_{0}\right)+2\sum_{k=1}^{p}\lambda_{k}\left[\sum_{i=1}^{n}\omega_{i}s_{k}\left(x_{i}\right)-s_{k}\left(x_{0}\right)\right]$$

By solving the partial derivatives *λ_k_* and *ω_i_*, we get equations of the Kriging weights:

(9) $$\{\begin{array}{c} \sum_{j=1}^{n}\omega_{j}Cov\left[Z\left(x_{i}\right),Z\left(x_{j}\right)\right]+\sum_{k=1}^{p}\lambda_{k}s_{k}\left(x_{i}\right)=Cov\left[Z\left(x_{i}\right),Z\left(x_{0}\right)\right],i=1,...,n\\ \sum_{i=1}^{n}\omega_{i}s_{k}\left(x_{i}\right)=s_{k}\left(x_{0}\right),k=1,...,p \end{array}$$

According to the solution to these equations, we can obtain the weights. The covariance is generally derived from the semivariogram model γ(⋅) which has the following form:

(10) $$\gamma \left(h\right)=\frac{1}{2}E\left[{\left(Z\left(x\right)-Z\left(x+h\right)\right)}^{2}\right]$$

where *Z*(*x*) and *Z*(*x* + *h*) are values of two points separated by vector ***h***. The semivariogram depends only on the vector ***h***. There are several ways to estimate the model. The classical formula is:

(11) $$\hat{\gamma }\left(h\right)=\frac{1}{2N\left(h\right)}\sum_{x_{i}-x_{j}=h}{\left(Z\left(x_{i}\right)-Z\left(x_{j}\right)\right)}^{2}$$

where *x_i_* and *x_j_* are a pair of two points separated by vector ***h***, and *N*(***h***) denotes the number of pairs.

#### 3.1.2. A Novel Method for Constructing the Semivariogram Models

**Figure 2:**
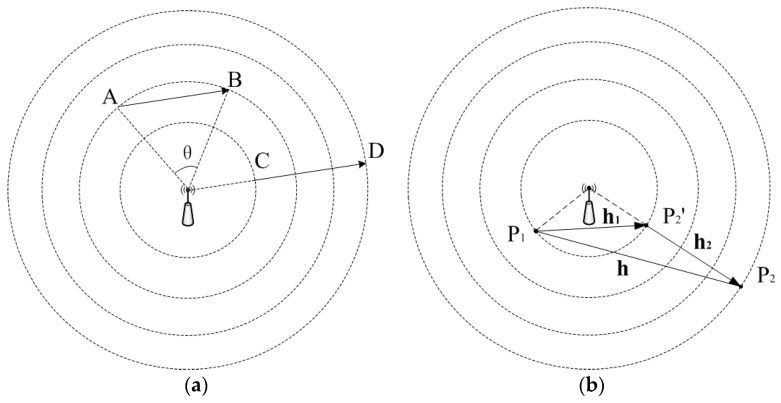
(**a**)Two pairs of data points A, B and C, D separated by two equal vectors $\vec{AB}$ and $\vec{CD}$ ; (**b**) the original vector ***h*** is decomposed into ***h***_1_ and ***h***_2_ according to the positions of the transmitter and two sampled points.

Conventional semivariogram model-based UK can certainly be used for RSSI estimation. However, by considering the characteristics of then spatial RSSI distribution, the performance may be improved. The spatial distribution of RSSI from an AP is similar to light waves spreading out from a point light source. Two pairs of data points that have equal separation distances may produce totally different variances. For example, in Figure 2a, two pairs of data points A, B and C, D are separated by vectors $\vec{AB}$ and $\vec{CD}$ which have equal norms and the same orientations. However, the RSSI variance between point A and B can be totally different from the counterpart between points C and D, since the resulting factors are different. Besides environmental factors, the two pairs of data points have their own specific factors. For points A and B, the main factor is the difference of attenuation directions of the radio signals. While for points C and D, the main factor is the difference of travel distances between radio signals. As we know, the impacts on RSSI caused by radio signals’ travel distances and attenuation directions are totally different, and if the RSSI variances caused by the two factors have not been treated discriminatively, the obtained semivariogram model will not be exact enough.

In this paper, we propose to decompose the original vector ***h*** between two data points into two vectors ***h***_1_ and ***h***_2_ according to the differences of attenuation directions and travel distances of two radio signals. In Figure 2b, two data points *P*_1_ and *P*_2_ are presented.

We have the original vector ***h*** ($\vec{P_{1}P_{2}}$). Suppose the spatial location of the transmitter is known, so we select *P*_1_ which is closer to the transmitter. Then a point *P*_2_’ that has the same distance to the transmitter as the selected point *P*_1_ can be found in the travel path of the radio signal passing through *P*_2_. Then we can get ***h***_1_ ($\vec{P_{1}P_{2}^{'}}$) and ***h***_2_ ($\vec{P_{2}^{'}P_{2}}$). Note that:

***h*** = ***h***_1_ + ***h***_2_ (12)

Following the above method for deriving two sub-vectors, we can obtain two semivariograms ${\hat{\gamma }}_{1}\left(h_{1}\right)$ and ${\hat{\gamma }}_{2}\left(h_{2}\right)$ from the original set of data points. Semivariogram ${\hat{\gamma }}_{1}\left(h_{1}\right)$ is with respect of the information of RSSI structure on the difference of local environments in the travel paths of radio signals, whereas semivariogram ${\hat{\gamma }}_{2}\left(h_{2}\right)$ represents the RSSI structure information on the difference of travel distances of the radio signals. The two semivariograms are built independently, and then they are combined as a nested structure $\hat{\gamma }\left(h\right)$:

(13) $$\hat{\gamma }\left(h\right)={\hat{\gamma }}_{1}\left(h_{1}\right)+{\hat{\gamma }}_{2}\left(h_{2}\right)$$

By the way, if the spatial location of the transmitter is unknown, we can still rely on the traditional method to obtain $\hat{\gamma }\left(h\right)$.

### 3.2. Differential RSSI-Based Location Estimation

#### 3.2.1. Eliminating the Impacts of Heterogeneous Reference Devices

In the proposed approach, RSSI is estimated by using the data measured by multiple reference devices. Since RSSI values are related to the configurations of the transmitter and receiver, it is reported that the value of the difference caused by device heterogeneity has a level of 10 to 20 dBm [33]. As a result, the estimated RSSI also contains heterogeneous measurement differences. We conducted an experiment to verify the difference between the estimated RSSI and observed RSSI at the same location. We used a mobile device to measure RSSI values from nine WLAN APs at a location. For each AP, we collected 100 samples, and then obtained the mean value of the RSSI values. That is the observed RSSI sample. At the same location, we also use the modified UK method to estimate RSSI values with respect to the nine APs.

**Figure 3:**
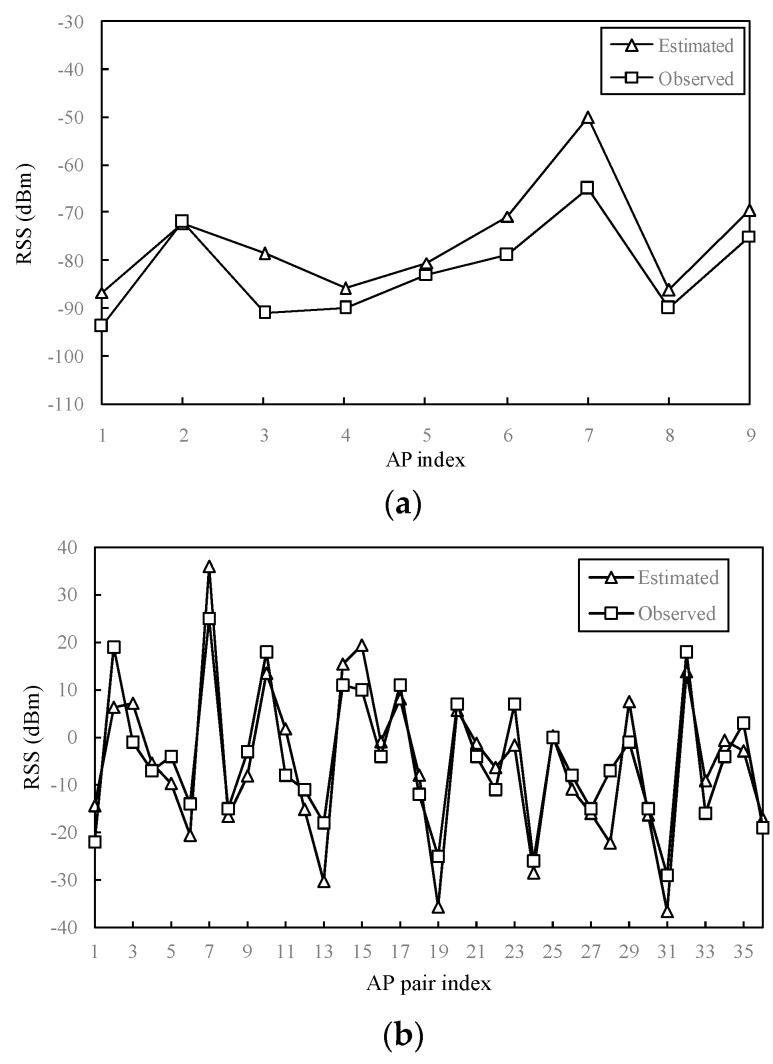
Observed values and estimated values by the modified UK from different APs (or AP pairs) at the same location. (**a**) Raw RSSI; (**b**) Differential RSSI.

Figure 3a presents the estimated and observed RSSI values with respect to the nine detected WLAN APs. Obviously, the more similar these two RSSI samples are the better. However, there are differences (8 to 10 dBm) between the estimated and observed values. Thus, the negative impacts of heterogeneous reference devices should be alleviated beforehand. Since it is hard to establish relationships between pair-wise devices for the proposed multiple reference devices-based solution, we apply the differential value metric which does not need a training process. According to [30], differential RSSI eliminates receiver gains, and therefore reduces the impact of device heterogeneity. Since the estimated RSSI is derived from the data from multiple reference devices, it is verified whether differential RSSI is suitable for the elimination of measuring differences in the estimated RSSI. According to Equation (8), estimated RSSI is a weighted sum of measured RSSI values by multiple reference devices. Suppose:

(14) $$RSSI_{i,j}=\sum_{t}k_{t}RSSI_{t,j}$$

where *RSSI_t,j_* denotes the RSSI measured by reference device *t* from AP*_j_*, and *k_t_* denotes the associated weight. Thus, differential RSSI between *RSSI_i_*_,1_ and *RSSI_i_*_,2_ has the form of:

(15) $$\Delta RSSI_{1,2}=RSSI_{i,1}-RSSI_{i,2}=\sum_{t}k_{t}\left(RSSI_{t,1}-RSSI_{t,2}\right)$$

We can observe from the above expression that, although the estimated RSSI is derived from the data measured by several reference devices, measuring differences are still reduced since the receiver gains are eliminated likewise in Δ*RSSI*_1,2_. As Figure 3b presents, differential values respectively derived from observed RSSIs and estimated RSSIs are plotted. Since nine APs are observed, 36 pairs of APs are used to calculate differential values. Compared with Figure 3a, observed differential RSSIs and the estimated ones at the same point appear more similar in Figure 3b. Thus in differential RSSI metrics, the degree of similarity can be represented more accurately.

#### 3.2.2. Location Estimation

Based on the estimated radio map, *K* Weighted Nearest Neighbors (*K*WNN) is used for location estimation:

(16) X^=∑i=1KXiwi∑i=1Kwi

where *X_i_* denotes the location of selected location fingerprint, *K* denotes the number of selected location fingerprints, and *w_i_* denotes the weight which is always derived from the inverse distance between two RSSI vectors (one is the online measurement, and the other is the related location fingerprint). For example, the inverse Euclidean distance:

(17) $$w_{i}=1/\sqrt{\sum_{j=1}^{N}{\left(RSSI_{i,j}-RSSI_{0,j}\right)}^{2}}$$

where *N* denotes the number of APs, *RSSI*_0,*j*_ denotes the online measurement from the *j*th AP, and *RSSI_i,j_* denotes the measurement in the location fingerprint from the *j*th AP. This kind of calculation of weight doesn’t consider measuring differences which may degrade localization accuracy of a system. In our adaptive location fingerprinting, radio maps are generated by multiple reference devices. And measuring difference becomes a crucial problem for location estimation. We apply the form of differential RSSI vector presented in [33]. Although it has $\left(\begin{array}{c} N\\ 2 \end{array}\right)$ elements (*N* denotes the number of the elements in original RSSI vector) which seems redundant since only (*N* − 1) of the elements are independent, it is free from the process of selecting a key element which is essential for generating a vector to contain independent elements only. Given the form of differential RSSI vector, the weight *w_i_* of the selected location fingerprint is still derived from the inverse Euclidean distance between two differential RSSI vectors:

(18) $$w_{i}=1/\sqrt{\sum_{j=1,k=2,j<k}^{N-1,N}{\left(\left(RSSI_{i,j}-RSSI_{i,k}\right)-\left(RSSI_{0,j}-RSSI_{0,k}\right)\right)}^{2}}$$

## 4. Experimental Evaluation

### 4.1. Setup

**Figure 4:**
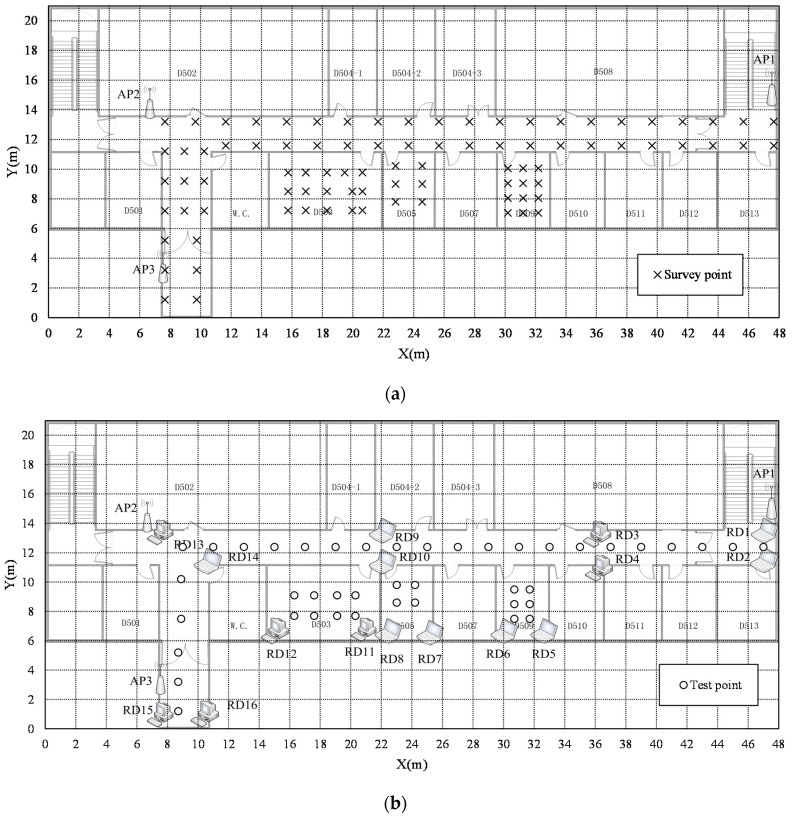
Layout of the test bed. (**a**) Spatial distributions of offline survey points; (**b**) Spatial distributions of multiple reference devices and online test points.

The proposed approach was evaluated on the fifth floor in a research building. The floor plan is shown in Figure 4.

The area measures 48 × 21 m^2^, and the test bed covers the whole corridor and three rooms. Sixteen PCs marked as RD1 to RD16 in Figure 4b are uniformly placed to play the roles of reference devices. There are 10 desktops, each of which is embedded with one of three types of IEEE 802.11 wireless network interface cards (WNICs): Netcore NW335, D-link DWA-123, and D-link DWL-G122. The others are laptops, two HP Mini 110s with Broadcom 802.11 b/g WNIC, two Lenovo E40s with Intel(R) dual band wireless-AC 3160 WNIC, one Acer Aspire 5100 with Atheros AR5005G WNIC, and one LenovoThinkpadSL400with Atheros AR5008 WNIC. In the experiments, the reference devices were activated to collect real-time RSSI data. A Dell Inspiron 14-7447 PC with Intel(R) Dual Band Wireless-AC 3160 was used as the mobile device. Using the mobile device, we collected test data during four time periods in two days for location calculation at 43 points marked by circles in Figure 4b. Details of the four time periods are as follows, one time period, 7 p.m. to 8 p.m. on the first day, and three time periods 10 a.m. to 11 a.m., 3 p.m. to 4 p.m., and 4 p.m. to 5 p.m. on the next day. At each point 100 samples were collected. To form an offline radio map, the mobile device was used to collect 88 location fingerprints at the survey points marked by crosses in Figure 4a. At each point we also collected 100 samples. All the devices have the same sample frequency (1 Hz). Tens of wild WLAN APs with unknown positions can be scanned in the area, and three more marked by AP1, AP2, AP3 are added. The wild APs may be on the same floor outside this test bed or on different floors, or even in different buildings. All the wild and added APs were used for positioning. However none of the APs covers the entire area, and at each sampling point 10 to 20 APs can be scanned on average.

To evaluate the proposed approach, two interpolants, IDW and Universal Krigingas well as the neural network (NN)-based approach proposed in previous work [4] are used as the baseline.Since location results based on the offline radio map are not so good which prevent those offline radio map-based adaptive approaches [3,4] from providing ideal location performances, we use the RSSIs estimated by our modified UK method to train the NN-based regression functions between each survey point and its neighboring reference devices. Default settings for all the experiments are as follows, the threshold forneural network training are 2000 times, the smoothing window size for the data used for RSSI estimation and location estimation is 20K in KWNN for location estimation equals 8.

### 4.2. Results and Analysis

**Figure 5:**
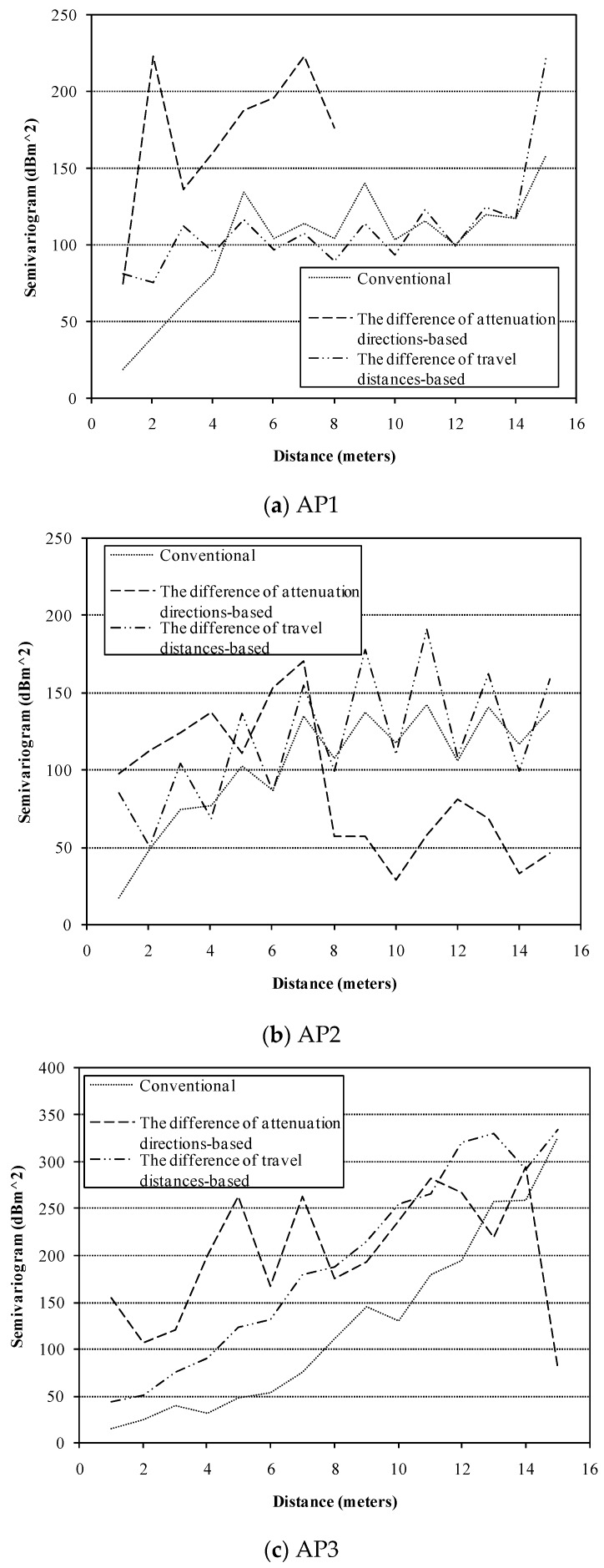
Semivariograms of different APs, (**a**) AP1; (**b**) AP2; and (**c**) AP3 obtained by using three different manners.

For Kriging interpolants, the semivariogram model should be determined first. In the following tests, we applied the models approximated depending on the collected offline radio map. Although the semivariograms are derived from the offline radio map, the two UK interpolants, conventional UK and the modified UK still produce accurate location results as presented in the following tests. This fact implies that the two UK interpolants both adapt to dynamic environments. As for each of the added APs, the semi-variances of RSSI values in the test area with respect to different manners were calculated and presented in Figure 5, respectively. Each curve for an AP was obtained by using a different manner, and it represents quantitative structural characteristics of RSSIs’ spatial distribution in the test area. We find that the obtained statistical results based on the difference of travel distances of radio signals are similar with the results based on the conventional manner. But the results based on the difference of attenuation directions of radio signals are somewhat different. It implies that a combination of semivariograms based on the difference of attenuation directions and the difference of travel distances of radio signals contains more information about the environments than conventional semivariogram, since the difference of attenuation directions of radio signals represents the difference of local environments in the travel paths of radio signals. And therefore, the combination can enhance the prediction of RSSI. To fit the experimental semivariogram, we choose the spherical model [40]:

(19) $$\gamma \left(h\right)=\{\begin{array}{cc} 0, & h=0\\ C_{0}+C\left(\frac{3}{2}\frac{h}{a}-\frac{1}{2}{\left(\frac{h}{a}\right)}^{3}\right), & 0<h\leq a\\ C_{0}+C, & h>a \end{array}$$

where *a* denotes range, C_0_ denotes nugget, and C_0_ + C denotes sill. γ(0) = 0 means a discontinuity at the origin, which is called the “nugget effect”. For each AP, the parameters C_0_ and C were obtained according to the approximated model, but the parameter *a* was assumed as a fixed value, 8 m.

**Figure 6:**
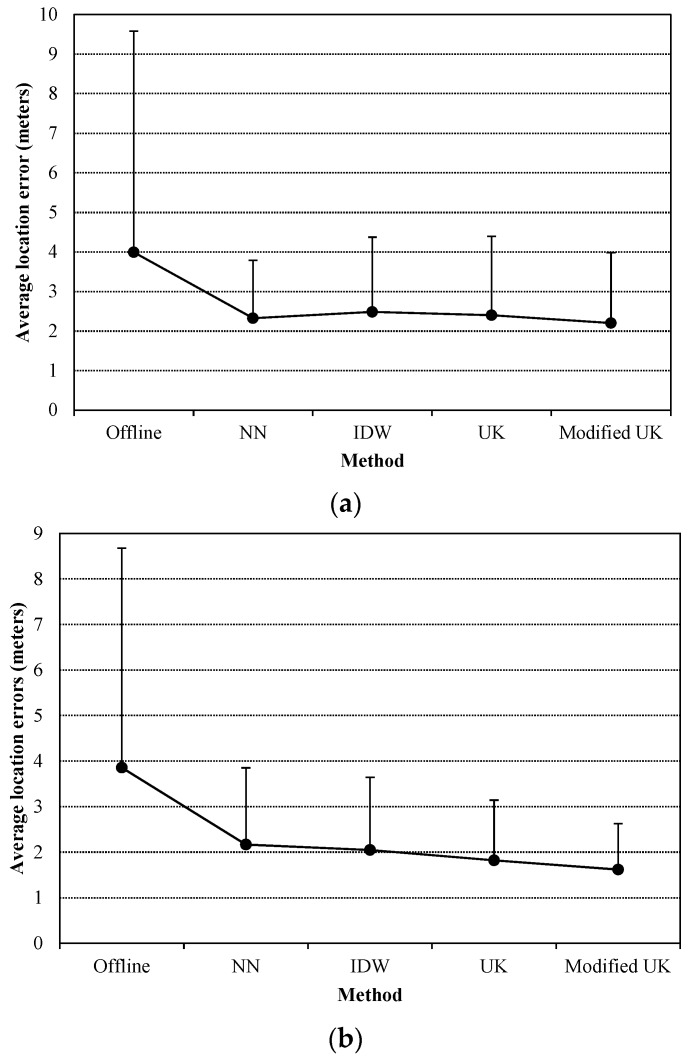
Average location errors (SDs of the location errors are used as the error bars) with respect to the offline radio map and the radio maps estimated by using the NN, IDW, UK, and the modified UK methods respectively in two time periods, (**a**) 7 p.m. to 8 p.m. in the first day and (**b**) 10 a.m. to 11 a.m. in the next day.

With the given parameters of UK interpolants, experiments were first conducted to evaluate the performances of the radio maps estimated by using real-time data against the offline radio map during two time periods, 7 p.m. to 8 p.m. on the first day and 10 a.m. to 11 a.m. on the second day. Figure 6 shows the average location errors and standard deviations (SD) of the location errors. All the real-time estimated radio maps provide better results in two time periods, compared to the offline radio map. It implies that it is possible to replace the offline radio map by real-time radio maps for practical use. Estimated radio maps-based average location errors are all around 2 m. And we can also observe that the modified UK performs comparable with the NN method, and performs better than the UK and IDW methods. As mentioned above, all the semivariogram models are derived from the offline radio map, and therefore the two UK interpolants adapt to dynamic environments.

**Figure 7:**
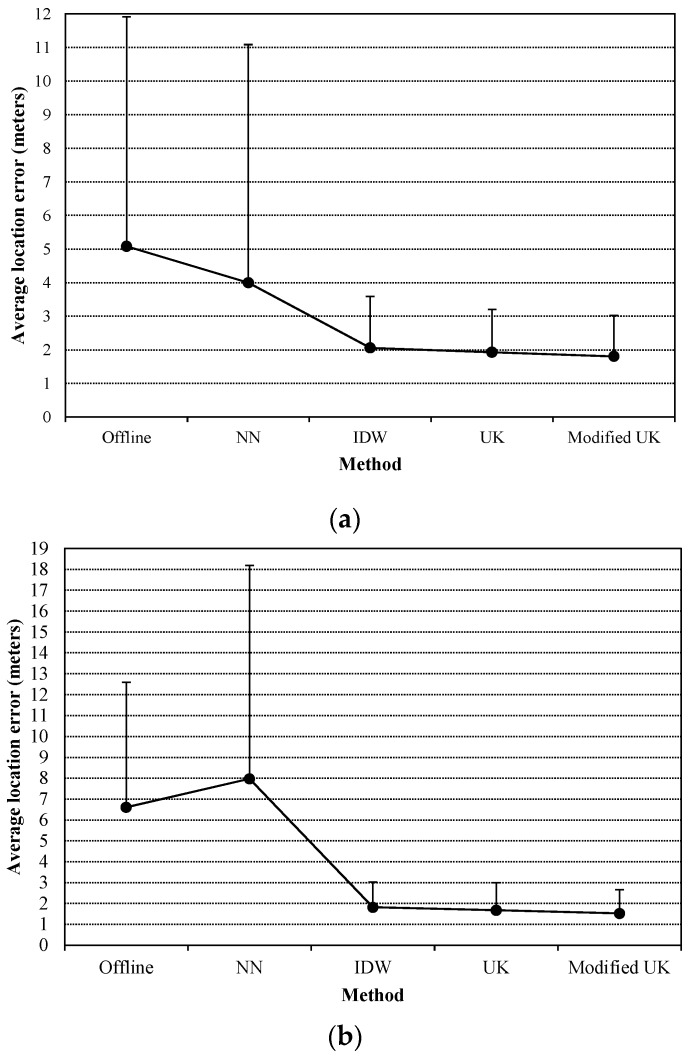
Average location errors with respect to the offline radio map and the radio maps estimated by using different approaches in two scenarios, (**a**) two APs’ positions are changed; (**b**) three APs’ positions are changed. In addition, SDs of the location errors are used as error bars.

Next, we tested the approaches in the situations of changing APs’ positions. The experiments were conducted during another two time periods, 3 p.m. to 4 p.m. and 4 p.m. to 5 p.m. The involved APs are the added ones. First, the positions of two APs were changed (we interchanged the positions of AP1 and AP2), and then three APs changed their positions (AP1 moves to the position of AP3, AP2 moves to the position of AP1, and AP3 moves to the position of AP2). Figure 7 presents the average location errors and SDs of the location errors. Since the test depicted in Figure 6b is conducted on the same day as the two tests here, and is without the impact of changing APs’ positions, it is used as the baseline for comparison. For the offline radio map and the NN-based method, compared with the location results presented in Figure 6b, the average location errors increase about 25% and 100%, respectively, when two APs’ positions are changed (as presented in Figure 7a), and increase over 60% and 300%, respectively, when three APs’ positions are changed (as presented in Figure 7b). This implies that the NN-based method cannot accommodate the change of APs’ positions. However, IDW and UK interpolants are flexible with the changing of APs’ positions and the average location errors with respect to the estimated radio maps by using the interpolants does not increase compared with the results presented in Figure 6b, which means that the conventional semivariogram model of an AP can still be used when the AP’s position has been changed. For the proposed method, the semivariogram models are associated with the positions of APs, but the changing of APs’ positions brings about little impact. Since compared with the distance from an AP to the sampling points, the distances between the sampling points are much shorter. And thus the impacts on the difference of attenuation directions and the difference of travel distances of two radio signals caused by the changing of APs’ positions are trivial.

**Figure 8:**
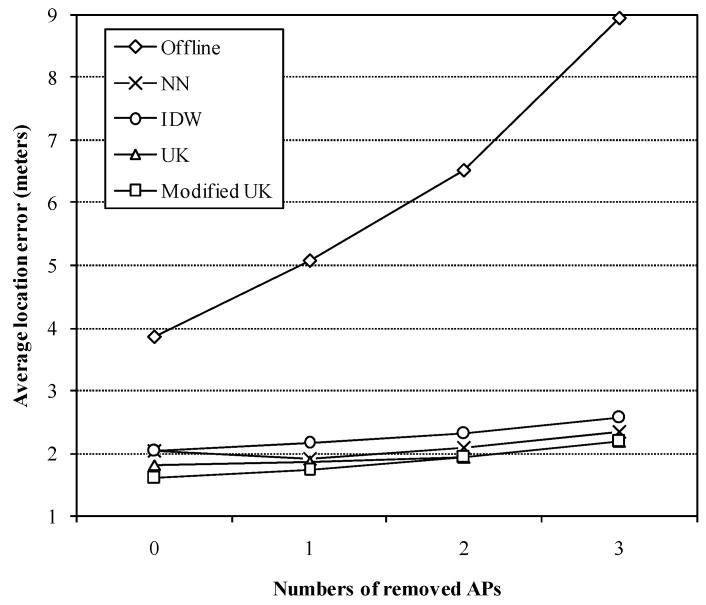
Average location errors of different approaches *versus* the numbers of APs.

In the following, we used the data collected during the time period from10 a.m. to 11 a.m. to test the approaches in cases of removing different numbers of APs. The involved APs are also the added ones. Figure 8 presents the average location errors. If the added APs are all removed (three APs are removed), the average location error based on the offline radio map increases significantly, but the estimated radio maps-based location accuracy still maintains high levels. And UK and the modified UK methods perform better than IDW method and the NN-based method in all cases. When the positions of more than two APs are known (one AP is removed or none is removed), the improvements of the proposed method in location accuracy are observable, compared with the conventional UK method.

**Figure 9:**
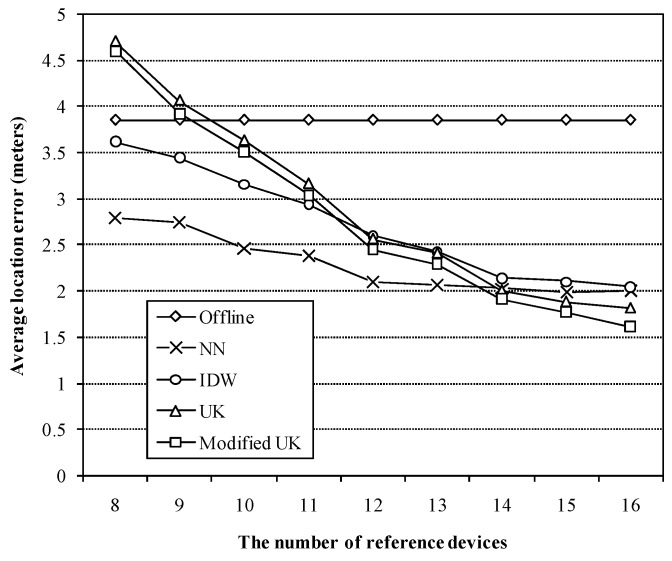
Average location errors of different approaches *versus* the number of reference devices.

Last but one, we tested the impact of the number of reference devices on the approaches based on the data during the time period, 10 a.m. to 11 a.m. To ensure that the left reference devices cover the entire test bed, we divided all 16 reference devices into eight groups, RD1 and RD2, RD3 and RD4, RD5 and RD6, RD7 and RD8, RD9 and RD10, RD11 and RD12, RD13 and RD14, as well as RD15 and RD 16, and make sure at least one reference device in each group is in operation during each test. Therefore, at least eight reference devices are required. Figure 9 presents the average location errors with respect to different numbers of reference devices.

For each number, we chose 40 random subsets of reference devices and compared the approaches. Since the offline radio map does not require any data from the reference devices, the average location error does not change with the number of reference devices. And the NN-based method is more robust than the three interpolants, IDW, UK and the modified UK with respect to the number of reference devices. Among the three interpolants, our proposed method performs better than conventional UK method as the number of reference devices decreases from 16 to 8. However, IDW method performs even better when the number of reference devices is less than 12. It implies that with lower density of reference devices, the interpolants should be combined with the NN-based method to provide reasonable estimation.

**Figure 10:**
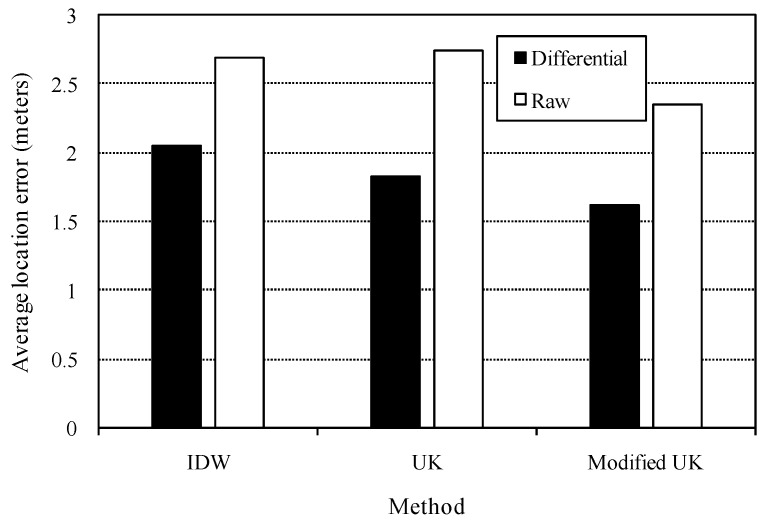
Average location errors of different approaches with respect to differential RSSI and raw RSSI metrics.

As for all the above tests, the estimated radio maps were derived from heterogeneous measurements, and therefore only differential RSSI was applied to location estimation. To reveal the superiority of differential RSSI metrics over raw RSSI metrics, comparative tests were finally conducted. The test data is again during time period from 10 a.m. to 11 a.m. Figure 10 presents the average location errors. We can observe that differential RSSI improves the location accuracy significantly. This implies that device heterogeneity causes severe negative impacts on multiple reference device-based adaptive location fingerprinting, and differential RSSI is efficient in this situation.

From the above experiments, we can conclude that the proposed approach not only adapts to dynamic environments and the situations of changing APs’ positions, but it is also robust with respect to reference device heterogeneity. However, it is less robust than regression function-based methods [3,4] with respect to the number of reference devices. Thus, a combination of the proposed approach and regression function-based methods would be the good future choice for adaptive location fingerprinting.

## 5. Conclusions

In this paper, we propose an adaptive location fingerprinting approach assisted by reference devices. The approach is not only robust for dynamic environments and the situation of changing APs’ positions, but it also accommodates measurement differences caused by reference device heterogeneity. Two issues are studied in the proposed adaptive location fingerprinting approach, RSSI interpolation in indoor areas, and measurement differences between heterogeneous reference devices. For the interpolation of RSSI, the modified UK is proposed and provides better results than conventional UK because it takes the characteristics of RSSIs’ spatial distribution into account. Differential RSSI is effective for the alleviation of the negative impacts caused by heterogeneous reference devices. Since the proposed approach is less robust than regression function-based methods [3,4] with respect to the number of reference devices, in the future it will be combined with those methods to achieve better location performance.

## References

[1] Bahl P., Padmanabhan N.V. RADAR: An In-building RF-based User Location and Tracking System. Proceedings of the 19th Annual Joint Conference of the IEEE Computer and Communications Societies.

[2] Lim H., Kung L., Hou J.C., Luo H. (2010). Zero-configuration indoor localization over IEEE 802.11 wireless infrastructure. Wirel. Netw..

[3] Yin J., Yang Q., Ni M.L. (2008). Learning Adaptive Temporal Radio Map for Signal-strength-based Location Estimation. IEEE Trans. Mobile Comput..

[4] Wang H., Ma L., Xu Y., Deng Z. Dynamic Radio Map Construction for WLAN Indoor Location. Proceedings of the 3rd International Conference on Intelligent Human-Machine Systems and Cybernetics.

[5] Wu D., Xia L. (2015). Dynamic Adaptive Model for Indoor WLAN Localization. Acta Geod. Cartogr. Sin..

[6] Ni M.L., Liu Y., Lau Y.C., Patil A.P. LANDMARC: Indoor Location Sensing Using Active RFID. Proceedings of the 1st IEEE Int’l Conference Pervasive Computing and Communications.

[7] Krishnan P., Krishnakumar A.S., Ju W., Mallows C., Gamt S.N. A System for LEASE: Location Estimation Assisted by Stationary Emitters for Indoor RF Wireless Networks. Proceedings of the 23rd Annual Joint Conference of the IEEE Computer and Communications Societies.

[8] Atia M.M., Noureldin A., Korenberg M.J. (2013). Dynamic online-calibrated radio maps for indoor positioning in wireless local area networks. IEEE Trans. Mob. Comput..

[9] Du Y., Yang D., Xiu C. (2015). A novel method for constructing a WiFi positioning system with efficient manpower. Sensors.

[10] Hossain A.K.M.M., Soh W. (2015). A survey of calibration-free indoor positioning systems. Computer Commun..

[11] Youssef M.A. (2004). HORUS: A WLAN-Based Indoor Location Determination System. Ph.D. Thesis.

[12] Kaemarungsi K., Krishnamurthy P. (2012). Analysis of WLAN’s received signal strength indication for indoor location fingerprinting. Pervasive Mob. Comput..

[13] Xiang Z., Song S., Chen J., Wang H.A. (2004). Wireless LAN-based Indoor Positioning Technology. IBM J. Res. Dev..

[14] Chen L., Li B., Zhao K., Rizos C., Zheng Z. (2013). An improved algorithm to generate a Wi-Fi fingerprint database for indoor positioning. Sensors.

[15] Chintalaoudi K., Iyer A.P., Padmanabhan V.N. Indoor localization without the pain. Proceedings of the 16th Annual International Conference on Mobile Computing and Networking.

[16] Kim Y., Shin H., Chon Y., Cha H. (2015). Crowdsensing-based Wi-Fi radio map management using a lightweight site survey. Comput. Commun..

[17] Kuo S., Tseng Y. (2011). Discriminant minimization search for large-scale RF-based localization systems. IEEE Trans. Mob. Comput..

[18] Talvitie J., Renfors M., Lohan E. (2015). Distance-based interpolation and extrapolation methods for RSS-based localization with indoor wireless signals. IEEE Trans. Veh. Technol..

[19] Krumm J., Platt J. (2003). Minimizing Calibration Effort for an Indoor 802.11 Device Location Measurement System, Technical Report of Microsoft. http://research.microsoft.com/pubs/68919/tr-2003-82.pdf.

[20] Chai X., Yang Q. (2007). Reducing the calibration effort for probabilistic indoor location estimation. IEEE Trans. Mob. Comput..

[21] Li B., Wang Y., Lee H.K., Dempster A., Rizos C. (2005). Method for yielding a database of location fingerprints in WLAN. IEE Proc. Commun..

[22] Jan S., Yeh S., Liu Y. (2015). Received signal strength database Interpolation by Kriging for a Wi-Fi indoor positioning system. Sensors.

[23] Liu Y., Jan S. Assessment of indoor positioning system using Kriging fingerprinting method and IEEE 802.11v standard. Proceedings of the 27th International Technical Meeting of the Satellite Division of the Institute of Navigation (ION GNSS+ 2014).

[24] Li J., Heap A.D. A review of spatial interpolation methods for environmental scientists. https://d28rz98at9flks.cloudfront.net/68229/Rec2008_023.pdf.

[25] Lee M., Han D. (2012). Voronoi Tessellation Based Interpolation Method for Wi-Fi Radio Map Construction. IEEE Commun. Lett..

[26] Fang S., Wang C., Chiou S., Lin P. Calibration-free approaches for robust Wi-Fi positioning against device diversity: A performance comparison. Proceedings of the IEEE 75th Vehicular Technology Conference.

[27] Tao P., Rudys A., Ladd A.M., Wallach D.S. Wireless LAN location sensing for security applications. Proceedings of the Second ACM Workshop on Wireless Security.

[28] Haeberlen A., Flannery E., Ladd A.M., Rudys A., Wallach D.S., Kavraki L.E. Practical robust localization over large-scale 802.11 wireless network. Proceedings of the 10th Annual International Conference on Mobile Computing and Networking.

[29] Tsui A.W., Chuang Y., Chu H. (2009). Unsupervised learning for solving RSS hardware variance problem in WiFi localization. Mob. Netw. Appl..

[30] Wang J., Gao Q., Wang H., Chen H., Jin M. (2011). Differential radio map-based robust indoor localization. EURASIP J. Wirel. Commun. Netw..

[31] Hossain A.K.M.M., Jin Y., Soh W., Van Nguyen H. (2013). SSD: A robust RF location fingerprint addressing mobile devices’ heterogeneity. IEEE Trans. Mob. Comput..

[32] Dietrich P.F., Gregg D.S. Enhanced wireless node location using differential signal strength metric. http://www.patentsencyclopedia.com/app/20110183688.

[33] Dong F., Chen Y., Liu J., Ning Q., Piao S. A calibration-free localization solution for handling signal strength variance. Proceedings of the Second International Workshop on Mobile Entity Localization and Tracking in GPS-Less Environments.

[34] Figuera C., Rojo-Alvarez J.L., Mora-Jimenez I., Guerrero-Curieses A., Wilby M., Ramos-Lopez J. (2011). Time-space sampling and mobile device calibration for WiFi indoor location systems. IEEE Trans. Mob. Comput..

[35] Laoudias C., Zeinalipour-Yazti D., Panayiotou C.G. Crowdsourced indoor localization for diverse devices through radiomap fusion. Proceedings of the International Conference on Indoor Positioning and Indoor Navigation.

[36] Kjaergaard M.B., Munk C.V. Hyperbolic location fingerprinting: A calibration-free solution for handling differences in signal strength. Proceedings of the Sixth Annual IEEE International Conference on Pervasive Computing and Communications.

[37] Cheng W., Tan K., Omwando V., Zhu J., Mohapatra P. RSS-Ratio for enhancing performance of RSS-based applications. Proceedings of the 32nd Annual IEEE International Conference on Computer Communications.

[38] Rappaport T.S. (2002). Wireless Communications: Principles and Practice.

[39] Cressie N.A.C. (1991). Statistics for Spatial Data.

[40] Goovaerts P. (1997). Geostatistics for Natural Resources Evaluation.

